# Genetic Polymorphisms and the Clinical Response to Systemic Lupus Erythematosus Treatment Towards Personalized Medicine

**DOI:** 10.3389/fphar.2022.820927

**Published:** 2022-03-18

**Authors:** Melisa Intan Barliana, Nadiya Nurul Afifah, Riezki Amalia, Laniyati Hamijoyo, Rizky Abdulah

**Affiliations:** ^1^ Department of Biological Pharmacy, Faculty of Pharmacy, Universitas Padjadjaran, Bandung, Indonesia; ^2^ Centre of Excellence in Higher Education for Pharmaceutical Care Innovation, Universitas Padjadjaran, Bandung, Indonesia; ^3^ Department of Pharmacology and Clinical Pharmacy, Faculty of Pharmacy, Universitas Padjadjaran, Bandung, Indonesia; ^4^ Department of Internal Medicine, Rheumatology Division, Faculty of Medicine, Universitas Padjadjaran, Hasan Sadikin Hospital, Bandung, Indonesia

**Keywords:** Genetic polymorphism, pharmacokinetics, pharmacodynamics, systemic lupus erthematosus, precision medicine

## Abstract

Systemic lupus erythematosus (SLE) is a chronic autoimmune disease characterized by a broad spectrum of clinical manifestations, an aberrant autoimmune response to self-antigens, which affect organs and tissues. There are several immune-pathogenic pathways, but the exact one is still not well known unless it is related to genetics. SLE and other autoimmune diseases are known to be inseparable from genetic factors, not only pathogenesis but also regarding the response to therapy. Seventy-one human studies published in the last 10 years were collected. Research communications, thesis publication, reviews, expert opinions, and unrelated studies were excluded. Finally, 32 articles were included. A polymorphism that occurs on the genes related to drugs pharmacokinetic, such as CYP, OATP, ABC Transporter, UGT, GST or drug-target pharmacodynamics, such as FCGR, TLR, and BAFF, can change the level of gene expression or its activity, thereby causing a variation on the clinical response of the drugs. A study that summarizes gene polymorphisms influencing the response to SLE therapy is urgently needed for personalized medicine practices. Personalized medicine is an effort to provide individual therapy based on genetic profiles, and it gives better and more effective treatments for SLE and other autoimmune disease patients.

## 1 Introduction

Systemic lupus erythematosus (SLE) is a chronic autoimmune disease characterized by a broad spectrum of clinical manifestations, an aberrant autoimmune response to self-antigens, which can affect organs and tissues, while organ involvement is unpredictable. There are several immune-pathogenic pathways of SLE, but the exact one is still not well known unless it is related to genetics (La Paglia et al., 2017).

In general, innate and adaptive immune systems play a role in the pathogenesis of SLE. Activation of toll-like receptor (TLR) is a part of the innate immune system, leading to the downstream pathway for producing pro-inflammatory mediators, such as Interferon (IFN). Another innate immune system that plays a role in the pathogenesis of SLE is NETosis, a program for the formation of neutrophil extracellular traps. Apart from innate immune systems, adaptive immune systems are played by T- and B-lymphocytes. T-cells in SLE show a distorted gene expression, leading to the production of several cytokines. T-cells induce the activation of autoreactive B-cells, and the production of cytokine leads to autoantibody production, a hallmark of SLE. Moreover, B-cells serve as antigen-presenting cells, creating a loop to activate T-cells that leads to autoimmunity (Vaillant et al., 2021).

The presence of clinical manifestation and comorbidities caused by treatment of SLE can increase disease burden and lead to different choices and responses to therapy. One of the manifestations that affect drug disposition is kidney involvement. Lupus nephritis (LN) or renal disease manifestation such as nephrotic syndrome, may affect the pharmacokinetic profile of therapy. It should be tested for and monitored and assessed every 3 months to detect early signs of kidney disease (Tang et al., 2010; Artim-Esen et al., 2014; Duarte-García et al., 2017; Fanouriakis et al., 2019). In addition, liver involvement on SLE which may occur around 25–50% of SLE patients (van Hoek, 1996), can also affect the pharmacokinetics profile. Drug metabolizing enzymes are primarily decreased due to loss of liver tissue caused by hepatic disease (Elbekai et al., 2004).

There are three major comorbidities of SLE according to the updated European League Against Rheumatism (EULAR); first, Antiphospholipid syndrome (APS), associated with thrombotic and obstetric complication, lead to dangerous clotting in arteries and veins (Conti et al., 2016; Taraborelli et al., 2016; Bhana, 2020). Second, infections. The treatment of SLE with long and high dose glucocorticoid and other immune-suppressant could be a risk factor for patients with developing infections. The third is renal involvement. Disease activity like severe leucopenia and the presence of renal disease also contribute to the development of infections (Zou et al., 2013; Singh et al., 2016; Taraborelli et al., 2016; Hiraki et al., 2017; Rúa-Figueroa et al., 2017). Related diseases such as LN and the presence of APS as well as related treatments such as glucocorticoid use may be considered regarding the increased risk of cardiovascular development (Magder and Petri, 2012; Gustafsson and Svenungsson, 2014; Ballocca et al., 2015; Wu et al., 2016). This is important to consider regarding the possibility that polymorphisms can lead to an increased risk of the severity and burden of the comorbidities.

The diversity of drug responses has been revealed since the completion of the Human Genome Project. A developing field called “personalized medicine” has adapted medical care like treatment decision-making to the genetic background of individuals (National Human Genome Research Institute, 2003). The most common variation in human DNA is Single-Nucleotide Polymorphisms (SNPs), which are a single substitution of nucleotides for another (Lander et al., 2001; Subramanian et al., 2001; Kurkó et al., 2013). SNPs that occur in genes related to pharmacokinetics and pharmacodynamics drugs mechanism could affect the response, effectiveness, resistance, and toxicity of drug (Calcagno et al., 2017; Zastrozhin et al., 2018; Xiang et al., 2020). This article summarizes gene polymorphisms and their effects on genes related to the pharmacokinetic and pharmacodynamic mechanisms of SLE therapy.

## 2 Materials and Methods

**FIGURE 1:**
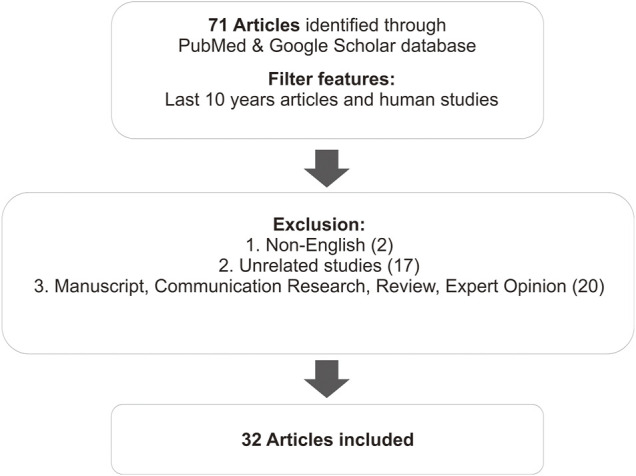
Research methodology flowchart.

This review summarizes the results of several studies related to the effects of polymorphisms on the therapy of SLE. It includes studies from the PubMed database identified using the keywords “genetic polymorphism,” “clinical response,” and “SLE therapy.” Articles that did not include therapy of SLE were excluded. Further, research communications, thesis publication, reviews, expert opinions, and unrelated studies were excluded (Figure 1).

Seventy-one human studies published in the last 10 years were collected. Then we conducted an abstract screening, analyzing to determine which articles were included in the inclusion criteria. Total 18 review studies, 2 thesis publications, and 17 unrelated studies, which discuss genetic polymorphism related to the susceptibility of SLE, were excluded. Hence, a total of 32 articles were included in this study. Most of the articles discuss the effect of gene polymorphisms on the therapeutic outcome—side effects, effectiveness, resistance, and survival rates–of the SLE therapy. All the data results of studies are summarized in a table, arranged by analyzed SNP, drugs used, and sample population. Differences in results between studies were discussed.

## 3 SLE Therapy

Based on updated 2019 EULAR recommendations for SLE management, SLE treatment aims to prevent organ damage, optimize health-related quality of life, and prolong the survival of patients. This can be achieved when organ/life-threatening SLE is treated early with the high-intensity immunosuppressive agent to curb disease activities, followed by a longer period of less intensive therapy to consolidate the response and prevent relapse. Prevention relapse was aimed at remission or lessening the disease activity, preventing flares in all organs, and treating by using the lowest possible dose of glucocorticoid for maintenance (Fanouriakis et al., 2019).

The first-line therapy for SLE is hydroxychloroquine (HCQ), known as the anti-malarial group (Fanouriakis et al., 2019). This drug is recommended for all patients with SLE unless its contraindicated, such as hypersensitive with 4-aminoquinolone derivatives, patients with serious heart problems, and low blood sugar (Saghir et al., 2021). The HCQ combined with Glucocorticoid (GC) become the standard care of SLE therapy. GC pulses of intravenous methylprednisolone high dose (250–1,000 mg per day) could provide immediate therapeutic effect and enable the use of a lower dose of oral form. For chronic maintenance, GC should be minimized, then slowly tapering off, thus finally could be withdrawn. Immunosuppressive therapies, such as methotrexate (MTX), azathioprine (AZA), cyclophosphamide (CYC), and mycophenolate mofetil (MMF), can be included in the initial therapy of organ-threatening disease (Fanouriakis et al., 2019). Other immunosuppressive agents, tacrolimus, are commonly used particularly for LN (Szeto et al., 2008). The immunosuppressive agent could be used for HCQ nonresponsive patients (with or without GC) or additional therapy for patients unable to reduce GC doses. Biologic agents, such as anti-CD20 rituximab (RTX), or anti-B-cell Activating Factor (BAFF), belimumab can be used for patients who do not respond to standard-care, or for patients with organ-threatening disease refractory/intolerance/contraindication to standard immunosuppressive agent (Fanouriakis et al., 2019). Anti-tumor necrosis factor (anti-TNF) α such as infliximab, adalimumab, and etanercept exerts both deleterious tissue-damaging effects mainly through its pro-inflammatory activities and beneficial activities by dampening aggressive autoimmune responses, but the outcome may vary, depending on timing and duration of treatment (Zhu et al., 2010).

## 4 Genetic Polymorphism Influence Therapy Responses: Pharmacokinetics Mechanism

**FIGURE 2:**
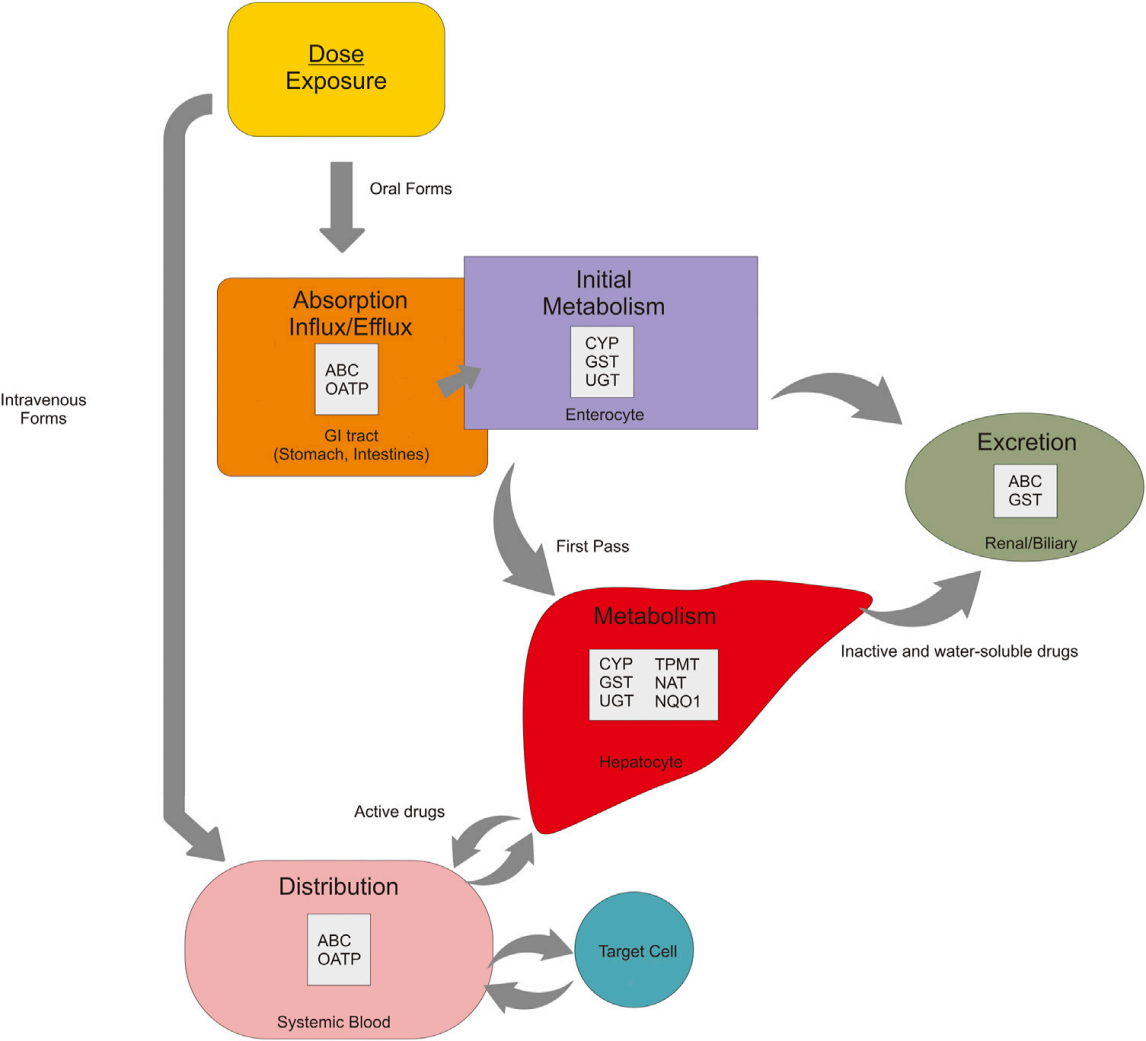
Pharmacokinetics mechanisms of the oral drug are affected by many proteins in every pathway—absorption, metabolism, distribution, and excretion.

The pharmacokinetics mechanism is divided based on the pathway: absorption, distribution, metabolism, and excretion (ADME) (Grogan and Preuss, 2020). In brief, the standard route of the drug journey explains the pharmacokinetics pathway. Through oral administration, absorption of the drug occurs in the lumen gut and then enters the liver to go through the first-pass metabolism, which activates the drugs. Besides the liver, drug metabolism occurs in the intestine (enterocyte) as an initial metabolism. When these drugs are active, they are distributed to target cells by blood circulation. On the target cells, drugs bind to the receptor resulting in molecular and physiological effects as pharmacodynamics mechanisms. After some time (half-life), the drugs begin to be eliminated from systemic blood, and these drugs return to the liver and get metabolized. The goal of this step is to make the drugs inactive and make the substrates ready to excrete. For intravenous administration, the drugs directly to the systemic circulation and then into the target cells (Doogue and Polasek, 2013). The fate of drugs in every pathway is influenced by proteins. SNPs of its encoded genes affect the function of these proteins, and thus the effect of the drugs (Figure 2).

### 4.1 Absorption, Distribution, and Excretion

#### 4.1.1 ABC Transporter Family (ABC)

ABC transporters cover a wide spectrum of substrates, including small inorganic and organic molecules (Wilkens, 2015). These transporters work via pumps and can move substrates in (influx) or out (efflux) of the cells. ABC transporters participate in the movement of most drugs and their metabolites across the cell surface and cellular organelle membranes; thus, defects in these genes can be important regarding cancer and autoimmune therapy, pharmacokinetics, and innumerable pharmacogenetic disorders (Vasiliou et al., 2009).

*ABCB1* rs2032582 and rs1128503 may influence the clinical efficacy of tacrolimus in patients with nephrotic syndrome (Li et al., 2018a). A study on *ABCC2* rs2273697 stated that Adenine-Guanine (AG) genotype is associated with lower MMF exposure (Yap et al., 2020). In contrast, a study of *ABCG2* in SLE patients, who use teriflunomide as treatment, showed that rs2231137 and rs2231142 were found to affect pharmacokinetics (Yao et al., 2019a; Yao et al., 2019b).

#### 4.1.2 Organic-Anion Transporter Polypeptide (OATP)

*OATP* gene encodes a sodium-independent organic anion transport system, which works on the basolateral (sinusoidal) membrane of hepatocytes for the uptake of certain organic anions (König and Fromm, 2011; NAKANISHI and TAMAI, 2012). OATPs are also involved in the intestinal absorption of drugs, expressed at the apical membrane of epithelial cells in the human small intestine (Kobayashi et al., 2003). OATPs protein is encoded by genes that are classified as solute carriers (SLC superfamily) (König and Fromm, 2011). A study on the effect of genetic polymorphism of transporter genes and associated functional alterations in drug transport showed that 12 *SLCO* transporter genes (OATP proteins) with different tissue expression profiles have been identified (NAKANISHI and TAMAI, 2012). Another study on autoimmune disease has a result that SLCO1B1 521T > C demonstrated a significant association with MMF, patients with Cytocine-Cytosine (CC) genotype showed a higher blood concentration level than the Thymine-Thymine (TT) genotype or the Cytosine-Thymine (CT) genotype (Shu et al., 2021). But, study on LN patients, MMF 12-h post-dose is related to renal flare, infection, and anemia, without significant association between genetic polymorphism of *OATP* rs7311358, and rs4149117, to the blood level of MMF (Yap et al., 2020).

### 4.2 Metabolism

#### 4.2.1 Cytochrome P450 (CYP)

CYP is a protein that plays a key role in the metabolism of drugs and other xenobiotic compound (Estabrook, 2003). Drug metabolism is categorized into two reaction phases—phase I and phase II. Drug metabolism is achieved through phases I, II, or both, catalyzed by the CYP system (Gibson and Skett, 2013). CYP450 was assigned a family number (e.g., CYP1, CYP2) and a subfamily letter (e.g., CYP1A1, CYP2D6). Several CYP proteins are found to be widespread throughout the body, demonstrating involvement in chemical activation, deactivation, and carcinogenesis (Estabrook, 2003). Genetic polymorphism of CYP exists, and the metabolism of certain drugs may be affected, varying the response of the therapy.

Many reports suggested that *CYP3A5* rs776746 has an impact on the clinical response of SLE regiments, such as tacrolimus and CYC. Studies in Japan show that Guanine-Guanine (GG) genotype (homozygote mutant) has higher blood concentrations of tacrolimus rather than in wild-type genotype (Ito et al., 2017). In that genotype, increasing blood concentration occurred in a whole subject (100%) (Yap et al., 2020). Another study on nephrotic syndrome subjects about tacrolimus response related to polymorphism at rs776746 showed no significant association (Li et al., 2018a). As well as studies in Korean SLE patients regarding HCQ response, polymorphism in *CYP2D6* influences the ratio of DCHQ: HCQ significantly (Lee et al., 2016). *CYP2C19* rs424485 is related to ovarian toxicity and risk of failure of CYC treatment (Ngamjanyaporn et al., 2011; Lee et al., 2016; Kumaraswami et al., 2017).

#### 4.2.2 Glutathione S- Transferase (GST)

GSTs are a family of detoxification (phase II metabolism reaction) enzymes that catalyze the conjugation of glutathione (GSH) to various endogenous and exogenous electrophilic compounds. It is divided into two super-family members, the membrane-bound microsomal and cytosolic. Microsomal GSTs influence the metabolism of leukotrienes and prostaglandins, whereas cytosolic GSTs are divided into six classes—α, μ, ω, π, θ, and ζ, and the π and μ classes play a regulatory role in MAP kinase (MAPK) pathway involved in cellular survival and death signals. Related to this role, GST has been implicated in the development of resistance toward chemotherapy agents (Townsend and Tew, 2003), and various members of the GSTs family member were found overexpressed in several patients (Allocati et al., 2018).

There are many studies on *Glutathione S- transferase Pi* (*GSTP1*) rs1695 with autoimmune subjects. In CYC treatment, SNP has a synergetic influence on CYC (Kumaraswami et al., 2017). Moreover, a decrease in activities of GSTP1 might be a background for more effective treatment of CYC (Hajdinak et al., 2020). *GSTA1* rs3957356 is associated with the risk of unresponsiveness and toxicity of CYC treatment (Attia et al., 2021).

#### 4.2.3 Arylamine N-Acetyltransferase (NAT)

NATs are polymorphic drug-metabolizing enzymes. The human gene products NAT1 and NAT2 have distinct substrate specificities: NAT2 is acetylated hydralazine, human NAT1 is acetylated p-aminosalicylate (p-AS) and the folate catabolite is para-amino benzoyl glutamate (p-abaglu). Human NAT2 is primarily in the liver and gut (Sim et al., 2014). The genetic polymorphism of liver *NAT2*, as an acetylator enzyme, causes individual variation in the response to a variety of amine drugs, such as teriflunomide (Spielberg, 1996; Hein et al., 2000). Some studies indicated the increasing frequency of slow acetylator phenotype in idiopathic SLE patients (Johansson et al., 1981; Spielberg, 1996; Hein et al., 2000), while other studies found no association (Kumana et al., 1990; Shiokawa et al., 1992). The observation of the drug-induced Lupus (DIL) found in the slow acetylator phenotype, suggests that non-acetylated drugs may accumulate and convert into reactive metabolites. Reactive metabolites might alter self-proteins presented to the immune system, thus stimulating T-cells which induce pathological and clinical signs of autoimmunity by different effector mechanisms (Griem et al., 1998; Zschieschang et al., 2002). *NAT* polymorphism could affect the pharmacokinetic profile of xenobiotic, risk of toxicity, and/or DIL, which means increasing the progression of SLE itself.

#### 4.2.4 UDP-Glucuronosyltransferase (UGT)

UGT enzymes play a key role in terminating the biological actions and enhancing the renal elimination of nonpolar (lipophilic) drugs from all therapeutic classes. Although the liver, a major detoxification organ has a vast abundance and diversity of UGTs, these enzymes exhibit significant but variable extrahepatic expression (Rowland et al., 2013). However, research conducted on LN with MMF treatment showed no association between UGT polymorphism with the clinical response of drugs, and the other studies have stated that there was no variation in genes in all study subjects (Yap et al., 2020).

#### 4.2.5 Thiopurine S-Methyltransferase (TPMT)

Thiopurine S-methyl transferase (TPMT) is a key enzyme involved in the metabolism of thiopurine drugs. Its function includes catalyzing the S-methylation of aromatic and heterocyclic sulfhydryl groups. Genetic heterogeneity of TPMT results in vast inter-individual-enzyme activity differences, that affect clinical efficacy and toxicity profiles (Weinshilboum and Sladek, 1980; Tai et al., 1996; Benkov et al., 2013; Coelho et al., 2016). Polymorphism of *TPMT* gene (rs1142345) on SLE patients with AZA is related to the risk of severe leukopenia and thrombocytopenia (myelosuppression toxicity) (Rashid et al., 2020).

#### 4.2.6 NAD (P) H Quinone Dehydrogenase (NQO)

NQO1 is one of the two major Quinone reductases in mammalian systems. NQO1 was hypothesized to influence the protection against oxidative stress and was shown to be a multifunctional antioxidant and an exceptionally versatile cytoprotector (Ross and Siegel, 2017). Genetic variations in *NQ O 1* can influence the expression of the genes known to play a central role in the glucocorticoid pathway, and increase the secretion of IL6 (a pro-inflammatory cytokine) (Maranville et al., 2011).

## 5 Genetic Polymorphism Influence Therapy Responses: Pharmacodynamics Mechanism

### 5.1 Fc Gamma Receptor (FCGR)

*FCGR* is a gene coding for Immunoglobulin G (IgG) Fc receptor that belongs to the immunoglobulin superfamily. FCGR is an essential receptor located in every cell surface of white blood cells. This receptor mediates the cellular effector function of IgG antibodies (Castro-Dopico and Clatworthy, 2019).

CD20 is a protein expressed on the surface of normal and malignant B-lymphocytes. An anti-CD20 monoclonal antibody can bind to FCGR and CD20, resulting in the linking of B-cell to its effector. Treatment with anti-CD20 antibodies can deplete all subsets of B-cells, except the early pre-B-cells and plasma cells. When B-cells are depleted, BAFF occasionally supports the survival and proliferation of B-cell. Polymorphism in *BAFF* has been discussed in the following paragraph (Boross and Leusen, 2012; Ajeganova et al., 2017). In contrast, the monoclonal antibody of TNF αn (anti-TNF α) binds to FCGR and the effector cells, then induces cell death. This mechanism is called antibody-dependent cellular cytotoxicity (ADCC). Another pathway called Complement Dependent Cytotoxicity (CDC) induces cell death by bonding protein C1q to a monoclonal antibody, resulting in the formation of a Membrane Attack Complex and target cell lysis (Boross and Leusen, 2012; Julià et al., 2013). Polymorphism on *FCGR* causes alteration of bond affinity between the monoclonal antibody and FCGR and affects the individual response to monoclonal antibody (Julià et al., 2013; Ajeganova et al., 2017).

Previous studies have analyzed the relationship between polymorphisms in *FCGR* and the clinical response of therapy. The majority shows a significant association between the presence of SNPs in *FCGR3A*, *FCGR2A*, and *FCGR2B* with clinical outcomes of anti-CD20 and anti-TNF-α. Some studies have stated that polymorphism of *FCGR3A* is associated with the improvement of clinical response and longer Progression-Free Survival (PFS) (rs10127939). Another study has stated that the V allele as a wild-type of *FCGR3A* rs396991 could be an indicator of biological therapeutic activity and longer flare-free survival. For *FCGR2A*, studies have stated that polymorphism on rs1801274 affects the outcome of anti-TNF in patients with SLE; however, in contrast, another study provided no significant results (Treon et al., 2011; Robledo et al., 2012a; Ajeganova et al., 2017).

### 5.2 Interferon Gamma (IFNG) and Interleukin (IL)

**FIGURE 3:**
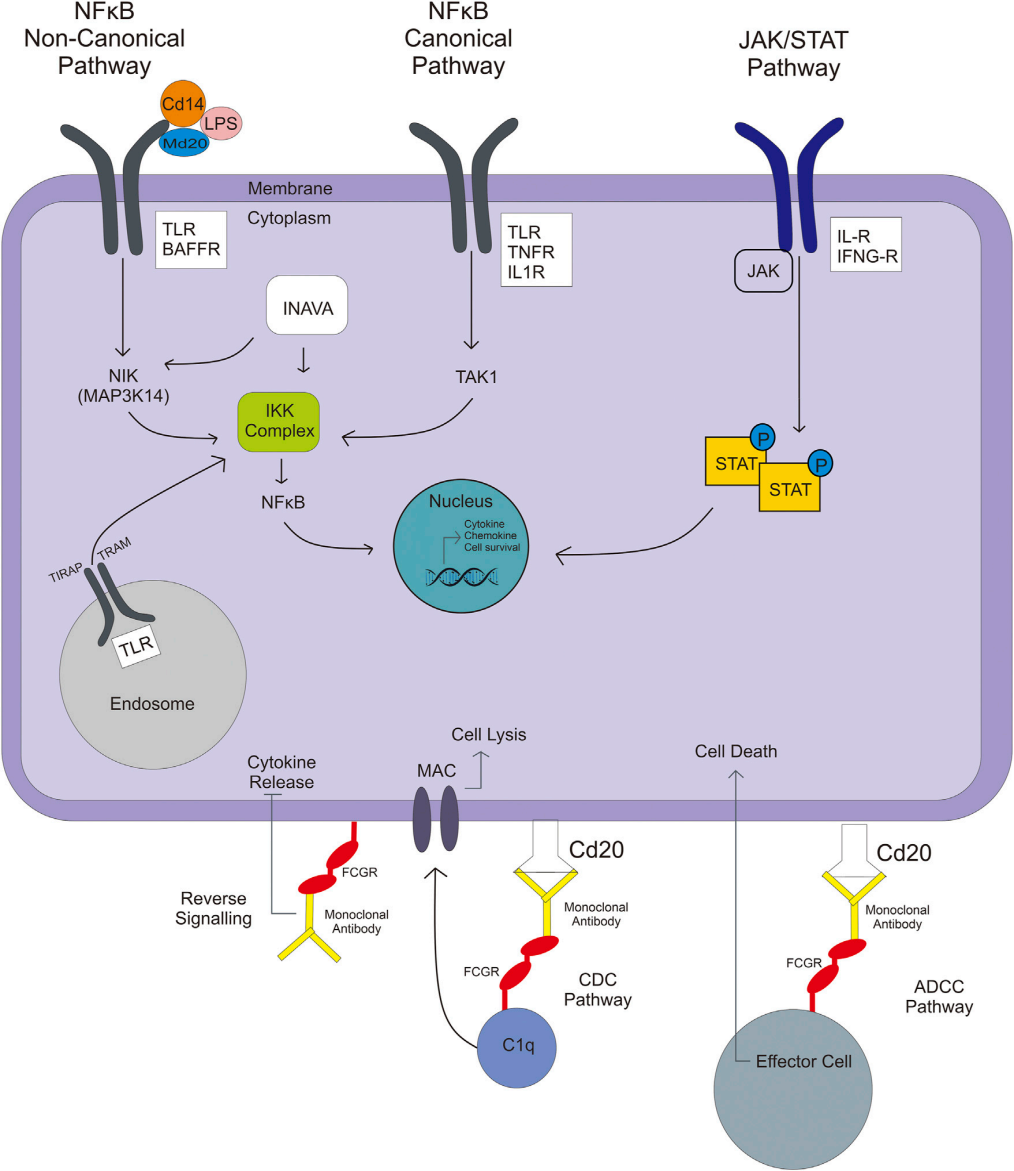
NFkB pathways involved in the activation of TLR or IL gene expression include noncanonical, canonical, and JAK/STAT pathways, which result in immunity responses like chemokine/cytokine production or survival cell.

IFNG plays a crucial role in innate and adaptive immune responses. IFNG is a pro-and anti-inflammatory cytokine. In addition to the role of IFNG in host defense, its excessive release has been associated with the pathogenesis of chronic inflammatory and autoimmune diseases (Mühl and Pfeilschifter, 2003). Interleukin is divided into three types, proinflammatory (e.g., IL-1, IL-17, IL-2, IL-21, etc.), anti-inflammatory (IL-10), and pro/anti-inflammatory (IL-6) (Zhang and An, 2007). Regulation of IFN-G and IL expression is largely driven by activators like transcription factors, and the expression of both cytokines could affect drug therapy (Zhang and An, 2007; Cuneo and Autieri, 2009). Methods to manipulate this upregulation involve specific systems using the phosphorylation of transcription factors, driven by activation of multimeric receptors of cytokines linked to Janus kinases (JAK) and signal transducer and activator of transcription (STAT) activation, called JAK/STAT pathway (Figure 3). When JAK is phosphorylated, it causes recruitment and phosphorylation of STAT. Then, phosphorylated-STAT will bind to another STAT and cause a homodimer, and then the homodimer STAT enters the nucleus and induces the transcription of genes involved in cell viability, survivability, or immunity. Another pathway related to regulating some interleukin (IL1) is the nuclear factor-kB (NFkB) pathway (Zhang and An, 2007; Bank et al., 2014).

A study in Denmark showed that *IFNG* polymorphism (rs2430561) may be an option for treating patients who do not respond to anti-TNF α^77^, whereas *IL-1*β genetic variance (rs1143623 and rs143627) related to increasing IL-1β levels may be unfavorable in treating psoriatic with anti-TNF α or ustekinumab (Loft et al., 2018). Glucocorticoid, treatment of several autoimmune diseases, influences clinical responses by stimulating the expression of genes that play central roles in IL-6 secretion (Maranville et al., 2011). SNPs rs6822844 (*IL-2* and *IL-21*) play a role in response to rituximab for treating SLE (Márquez et al., 2013). Lastly, the expression of IL-10, influenced by polymorphism rs1800896, affects the antimalarial regulation (downregulated) and leads to alteration of the clinical response (López et al., 2006).

### 5.3 TLR, Toll/Interleukin-1 Receptor Domain-Containing Adapter Protein (TIRAP), TNF, and BAFF

NFkB is a transcriptional regulator that deals with DNA transcription, cytokine and chemokine production, survival cell, and antibacterial production. NFkB activation has two types of pathway—canonical and noncanonical (Figure 3). Firstly, in a canonical pathway, lipopolysaccharide (LPS), TNFα, and IL-1 ligands are involved. The ligand binds to their receptor (TLR, TNFR, and IL-1R), which then activates the inhibitory kB kinase (IKK) complex. The IKK complex is a kinase that phosphorylates the IKKβ, forming IkB phosphorylated-NFkB complex. NFkB enters the nucleus, whereas ubiquitinated-IkB degrades by the proteasome. In contrast, in a noncanonical pathway, ligands that are involved are BAFF, leukotriene, CD40, and some of the LPS; LPS make a complex with CD14 and MD2/LY96. These ligand binds to their receptor (BAFFR, LTR, CD40), activating NFkB-inducing kinase (coded by *MAP3K14*), which leads to the IKK complex. When NFkB reaches the nucleus, it gets involved in the transcription process, and the result can be an immunity response like chemokine/cytokine production or survival cell (Lawrence, 2009; Bank et al., 2014).

Studies on relations between polymorphism in *TLR* gene and the clinical response of anti-TNF α, in psoriatic, Crohn’s disease, and ulcerative colitis patients showsative.

That the expression of the *TLR* gene is associated with the response of anti-TNF α. These studies stated that polymorphism in genes involved in activating the NFkB pathway is an important predictor of the clinical response of anti-TNF α (Bank et al., 2014; Loft et al., 2018). Moreover, the *TIRAP* gene was associated with a response to ustekinumab (Loft et al., 2018). The expression of TNFα, together with IL-10 expression, influenced by polymorphism of *IL-10* rs1800896, affects the antimalarial regulation (downregulated) and leads to alteration of the clinical response (López et al., 2006). Although TNFα agents such as infliximab are not included in the SLE treatment regimen in the updated EULAR, because of TNFα is known as Drugs-Induced Lupus (DIL), a case report of the use of TNFα in SLE patients with Ulcerative Colitis (UC) or Crohn’s disease (CD) resulted in no flare-up of SLE with CD remission, and modest efficacy was observed especially in patients UC with lupus nephritis, and no infliximab-induced SLE exacerbations were observed (Kearsley, 2000; Aringer et al., 2004; Principi et al., 2004; Aringer et al., 2009; Ben-Horin et al., 2016).

BAFF is a cytokine expressed mainly by neutrophils and monocytes. It plays a central role in B-cell proliferation, differentiation, survival, and immunoglobulin secretion. BAFF binding to BAFFR activates several downstream pathways that regulate essential survival functions, including protein synthesis and energy metabolism required to extend the half-life of immature, transitional, and maturation of B cells (Smulski and Eibel, 2018). Increases in BAFF in blood levels cause stimulation of B-cell production. As mentioned before, anti-CD20 monoclonal antibodies work to deplete B-cells by binding with CD20 and effector cells or C1q. Thus, the alteration in BAFF expression may affect the efficacy of monoclonal antibodies, especially anti-CD20 (Ajeganova et al., 2017). A similar pathway was happening with TLR, TNF, and IL-1R. Genetic polymorphism in gene influences the expression of the protein receptor, affecting the response of drug that works in NFkB or JAK/STAT pathway (Bank et al., 2014; Loft et al., 2018).

### 5.4 Innate Immunity Activator (INAVA/C1orf106)

INAVA is a protein that has a bacterial clearance function *via* Reactive Oxygen Species (ROS), autophagy, and Reactive Nitrogen Species (RNS). INAVA contains three 14-3-3 binding domains, and 14-3-3τ recruitment, in turn, modulates the recruitment of additional signaling molecules, including phosphorylated-extracellular signal-regulated kinase 1/2 (p-ERK), p-p38, and p-IkBα, which then contribute to the activation of downstream signaling pathways (MAPK and NFkB), followed by cytokine secretion as a final result of transcription in the nucleus. INAVA could be activated by TLR-induced cytokine or NOD2 stimulation. Polymorphism could make some alteration in INAVA expression and consistently decrease MAPK/NFkB signaling, and thus affect the cytokine secretion and bacterial clearance (Yan et al., 2017).

A study on INAVA polymorphism on autoimmune disease subjects treated with glucocorticoid shows a result that it has a significant local effect on GC response therapy; the polymorphism influence the expression of the gene known to play a central role in GC-related pathway (Maranville et al., 2011).

### 5.5 Autoimmune Regulator (AIRE)

Autoimmune regulator (AIRE) is a protein encoded by the *AIRE* gene, a potent repressor of autoimmunity, and it can cause severe autoimmune disease when it mutates. AIRE functions as a transcription regulator that promotes central immunological tolerance by inducing the ectopic thymic expression of many tissue-specific antigens. AIRE plays a role in removing autoreactive T-cells in the thymus. AIRE also plays a role in B-cell mediated immune response (De Martino et al., 2016). Alteration in expression of AIRE by downregulation is associated with drug response, especially in glucocorticoid treatment. A study on Negroid and Caucasians with inflammatory-autoimmune disease who were undergoing glucocorticoid treatment showed that *AIRE* polymorphism plays an important essential role in GC-related biological processes and manifests to the response of its therapy (Maranville et al., 2011).

### 5.6 TMEM 245 (C9orf5) (Transmembrane Protein - 245)

It is related to Microtubule Associated Protein causing an increase in mitotic delay and cell viability. TMEM245 is encoded by *C9Orf5*, which is distributed broadly across the mitotic spindle and is reversibly accumulated during reversible mitotic arrest (Liu et al., 2009). When the gene is upregulated and the expression of a protein encoded by the gene is increased, it could affect drug therapy like a better response. In glucocorticoid treatment, viable cells can enhance therapeutic response (Maranville et al., 2011).

### 5.7 Glucocorticoid Receptor (GR), Heat Shock Protein (HSP) and TNF Receptor-Associated Protein (TRAP)

**FIGURE 4:**
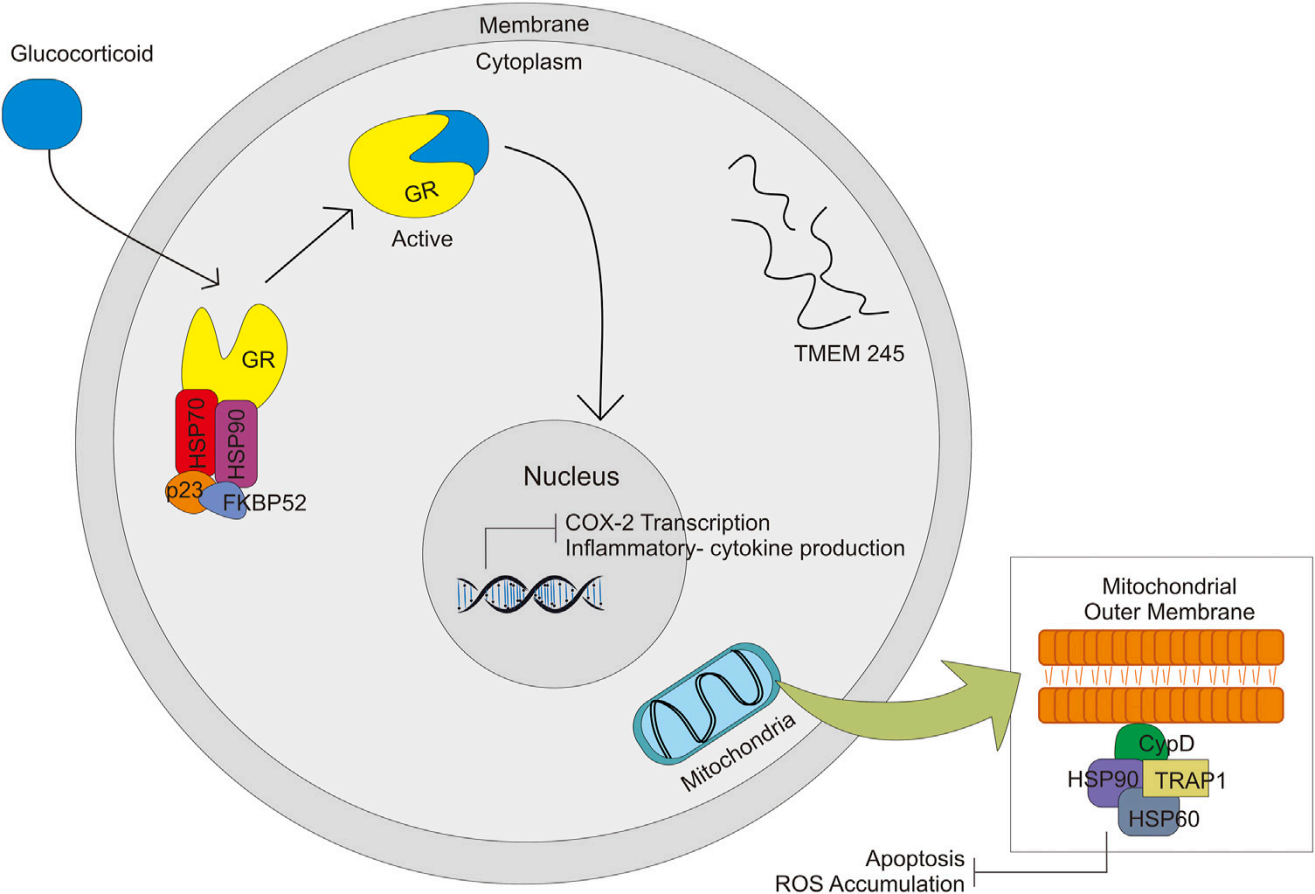
Signal pathway activated by GC through complex formation with HSP70, HSP90, p23, FKBP52, and GR. GC-GR complex formation will activate other signals in the nucleus and mitochondria.

GR, HSP90, and HSP70 with FKBP52 and p23 make a complex and activate the GR-glucocorticoid complex and then enter the nucleus, which leads to transactivation (Figure 4) (Oakley and Cidlowski, 2013). Diminished GR levels (mutation, variants, and low expression) could be a significant factor in glucocorticoid resistance. Another factor of Glucocorticoid resistance is an alteration of the function of GR-associated protein, like chaperons protein and nuclear factor. The HSP90, GR chaperone protein, influences the efficacy of Glucocorticoid (Leung and Bloom, 2003). A study on *GR* polymorphism (rs4912905, rs17100234, and rs7701443) in patients with SLE treated by GC showed that SNP affects the response of GC in SLE treatment (Zou et al., 2013). In contrast, a study related to *HSP90B1* polymorphism showed that polymorphism in the *HSP* gene might be associated with GC efficacy but with Health-Related Quality of Life (HRQoL) (Sun et al., 2018).

TRAP1 is a mitochondrial survival protein, belonging to the HSP90 family. Together HSP60 is physically associated with cyclophilin D (CypD), which is a physical component of the organelle permeability transition pore (PTP) (Altieri et al., 2012) (Figure 4). There is a general understanding that opening of the mitochondrial PTP is a key molecular for induction of mitochondrial apoptosis which leads to cell death (Green and Kroemer, 2004). The function is similar to HSP90 to some extent—protecting cells from oxidative stress damage through the fold and refolds damaged proteins. *TRAP1* gene is also thought to be associated with multidrug resistance. Overproduction of TRAP1 could reduce ROS accumulation, whereas its knockdown increases ROS formation. Excessive ROS leads to mitochondrial dysfunction, reduced viability of the cell, and cell death. Alteration in the expression of this gene might be associated with glucocorticoid response (Li et al., 2018b).

## 6 Discussion

**FIGURE 5:**
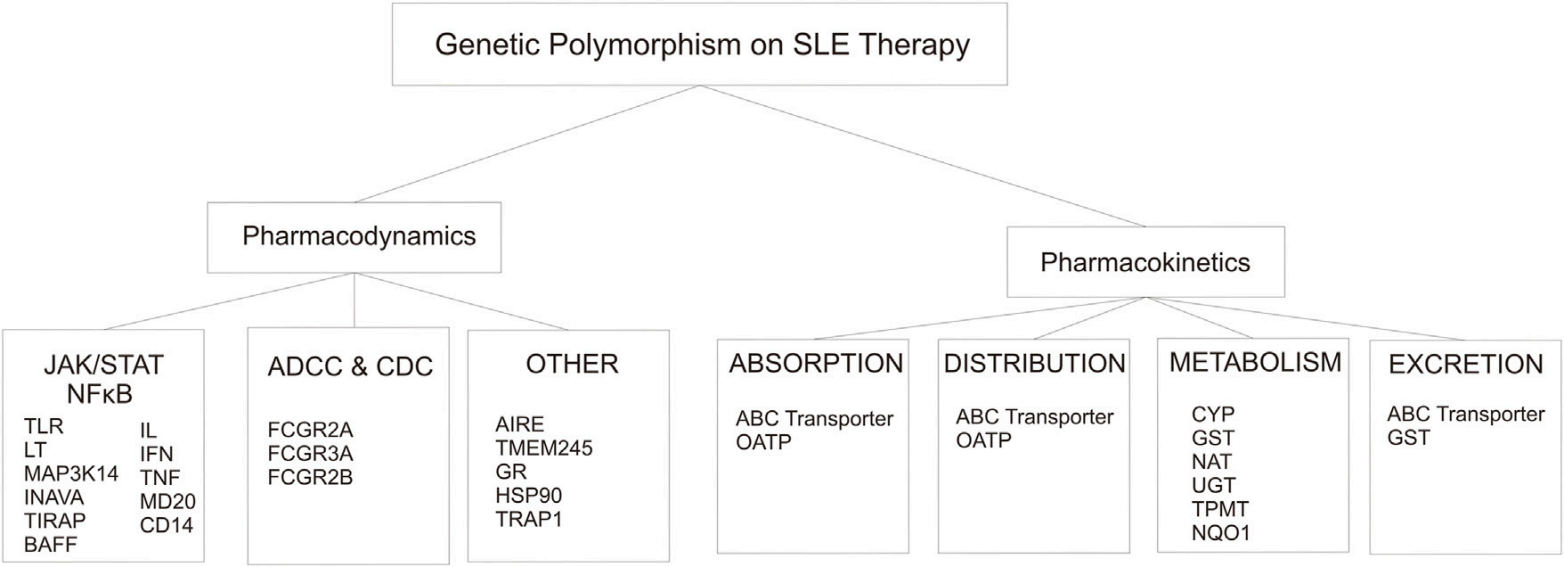
Genetic Polymorphism that involved in the alteration of pharmacodynamics and pharmacokinetics on SLE therapy.

Response to drug therapy varies among individuals. Variation in the human genome, such as the presence of SNPs, influences the disposition of drugs including pharmacokinetics (drug metabolism enzyme and transporter protein) (Supplementary Table S1) and pharmacodynamics (drug-target proteins) mechanism, thereby impacting drugs response (Supplementary Table S2) (Kitts et al., 2002; Lam, 2018). As we know that SLE and other autoimmune diseases can not be separated from genetic factors, which is affected the pathogenesis pathway and response of therapy. Polymorphism related to pharmacokinetics mechanism will affect drug availability at the target site, whereas polymorphism related to pharmacodynamics mechanism may affect individual sensitivity against drugs (Lam, 2018). The pharmacokinetic mechanism includes ADME is affected by several proteins (Figure 2). Genetic polymorphism on the gene encoding those proteins have been discovered that affects the expression of the gene, substrate specificity, intrinsic transport activity, or both (Lam, 2018). Mapping and finding out all of the SNPs related to the response of drug therapy used in SLE is the key to the success of autoimmune treatment as it can reduce or even eliminate the drug-related problems (adverse drug reactions/ADRs) and increase the efficacy of therapy (Figure 5).

The association of SLE with other diseases such as nephrotic syndrome, psoriasis, and ulcerative colitis, can be explained in terms of the pathophysiology and clinical manifestation of SLE. The therapeutic agent used between the disease also has similarities. So that, the genetic polymorphism that occurs on those diseases related to the therapy response can be taken into consideration in choosing therapy for SLE patients. The data showed that there was a potential risk of SLE patients will develop a LN within 3 years after the first onset, while 10–30% of LN have the potential to develop nephrotic syndrome or kidney failure (Ortega et al., 2010; Musa and Brent, 2021). Meanwhile, in the case of psoriasis, the pathologic mechanisms between SLE and psoriasis are different. But some case-study state that they experience a case of psoriasis accompanied by SLE. In addition, retrospective study results showed that the prevalence of psoriasis in SLE patients is greater than in the general population (Kim et al., 2015; Bonilla et al., 2016). Association between SLE and Ulcerative Colitis (UC), and Inflammatory Bowel disease (IBD), not known clearly. As well as the co-existence between SLE and UC. But, there is some case-report study about the co-existence between them. Besides, a study revealed that patients with SLE have a greater prevalence of IBD, which is also thought to be caused by gastrointestinal involvement of SLE (Koutroubakis et al., 1998; Shor et al., 2016; Mansour et al., 2018; Sun et al., 2019). RA features were common in SLE, as well as the personal history of the certain immune-related disease was strongly associated with increased risk of Waldenström Macroglobulinemia (Icen et al., 2009; Kristinsson et al., 2010; Pabón-Porras et al., 2019). SLE is also frequently complicated with cytopenias, including immune-trombocytopenia (Galanopoulos et al., 2017).

Cytochrome P450 super-family isoenzymes represent the most critical metabolic enzymes (Zanger et al., 2008). In general, which also works on SLE therapy, polymorphisms in the CYPs result in a phenotype alteration related to drug response that is categorized into; 1) the poor metabolizers (PMs), which exhibit abolished-enzyme activity, 2) the intermediate metabolizers, which reduce the activity of the enzyme, 3) the normal metabolizers or the extensive metabolizers, and 4) the ultrarapid metabolizers (UMs), which have high enzyme activity. For most drugs, PMs would exhibit a higher risk of ADRs. A slow rate of metabolism could affect the rate of drug elimination, increasing the concentration of the drugs trapped at the target site or the excretion organ. Thus, the toxicity of the drugs might be increased due to a poor metabolizer. A study on SLE patients with *CYP3A5* polymorphism (rs776746) to the tacrolimus response, showed that the phenotype of the polymorphism classified into PMs so that there was an increase in drug level on the mutant homozygote compared to the wild type (Ito et al., 2017). Therapeutic monitoring, selection of other drugs especially for patients who have a renal disease, and adjustment dose would be necessary for this case. A study stated that the mutant genotype was at a significantly higher risk of chronic irreversible drug-induced nephrotoxicity (Orlando et al., 2017). In contrast, UMs would experience lower efficacy when administered at a standard-dosage regimen of a drug, but it mostly depends on the polymorphic enzyme for elimination. This can be explained by the faster the metabolic rate; the drug will quickly turn into an inactive form and be prepared for elimination. One example of this case is from the study on rs776746 to the cyclophosphamide (CYC) drugs (Table S1). SNPs significantly decreased in AUC, C-max, and half-life; thus, patients with this polymorphism have a synergetic influence on CYC failure (Kumaraswami et al., 2017). In contrast, in the case of a prodrug, the UMs exhibit a higher incidence of ADRs because of the high concentration of drugs in their active form. Lastly, the PMs experience lower efficacy, reflecting a difference in the extent of therapeutically active metabolite formed between the two metabolic genotypes (Lam, 2018).

Other metabolic enzymes apart from CYPs (non-CYPs enzymes), such as UGT, GST, TPMT, NQO1, and NAT also play a role in influencing the metabolism and elimination rate of many drugs used on SLE. UGT contributes ∼35% of phase II drug metabolism. Polymorphism in *UGT* on LN patients who received kidney transplantation is known to affect the MMF exposure, with alteration in transactional and enzymatic activities (Table S1). MMF exposure can also be confounded by the use of GC high-dose. Therefore, the use of MMF and GC high-dose would be better delineated when a patient is on stable disease (Fleming and Weimert, 2010; Yap et al., 2020). In contrast, other studies on MMF observed no significant association (Yap et al., 2020). The variation in these results proves the need for further research for each drug against *UGT* polymorphism.

GST is a detoxification enzyme for the substrate, which includes carcinogens and chemotherapeutic agents including immunosuppressive agent, which is also used for SLE treatment (Lam, 2018). Decreased activity of GSTs could be a background of effective treatment or rather might be related to severe drug-related toxicity (McLeod et al., 2010; Hajdinak et al., 2020; Attia et al., 2021). A study on LN patients with *GSTP1* rs1695 polymorphism observed that heterozygote genotype has a better percentage of disease remission (*p* = 0.03) (Hajdinak et al., 2020), whereas another study on the same SNPs showed that rs1695 has a synergetic influence on CYC failure (Kumaraswami et al., 2017). That can be a consideration when choosing CYC as a treatment. In contrast, genetic polymorphism in *NAT* will affect their phenotype, and thus their enzyme activity. A study on SLE patients with teriflunomide (pyrimidine synthesis - inhibitor drug class–one of the NAT substrate) therapy, showed the SNP did not fulfill the relative standard error, representing the uncertainty of the effect (RSE <47%) (Lam, 2018; Yao et al., 2019b) (Table S1). So, NAT polymorphism has not been proven strong enough as a factor that needs to be considered in SLE treatment.

TPMT plays a major role in the inactivation of thiopurine drugs, including thioguanine, 6-mercaptopurine, and its precursor-like azathioprine. Previous studies on thiopurine with *TPMT* polymorphism show that *TPMT* mutant alleles have much higher cytotoxic thiopurine nucleotides and are at higher risk for developing severe hematological toxicities during treatment (Relling et al., 1999). A recent study on patients with SLE who were treated with azathioprine showed the same result, i.e., heterozygote genotype (rs1142345) at a risk of azathioprine induced myelosuppression (Rashid et al., 2020) (Table S1). A polymorphism that is associated with an increased risk of adverse effects, should be given caution especially if the patient has a comorbid related to it.

Pharmacodynamics mechanisms were divided into three groups based on the mechanism of action of the drugs on the SLE regiment (Figure 5). The first mechanism of action of the drugs involves intracellular signaling, such as JAK/STAT and NFkB pathway. The second is ADCC and CDC pathway. Last is the other group that includes the GC pathway, central tolerance mechanism, and mitosis.

Pharmacodynamics encompasses drugs, biochemical and physiological effects on the body, and the relationship between concentration and effect of the drug. Thus, every polymorphism that occurs in the genes related to the pharmacodynamics mechanism can have significant consequences for drug response by affecting either the activity or the expression of a drug-target or intracellular signaling protein (Lam, 2018).

For example, polymorphism on the *BAFF* gene will influence the expression of BAFF protein and then manifest to the serum level of BAFF (Cambridge et al., 2006) (Table S2). As the result of the studies that have been conducted on polymorphism in the *BAFF* gene, the T allele (mutant allele) has a positive association with the increased level of BAFF in serum. The heterozygote (CT) genotype of *BAFF* tends to cause a longer-progression-free survival, which might be related to the better outcome therapy than that of the homozygote (TT) (Ajeganova et al., 2017). Besides affecting protein expression, polymorphisms can affect the activity or bond affinity between drugs and their receptors, as in polymorphisms in the *FCGR* gene. Polymorphism on *the FCGR* gene influences the expression of FCGR and also controls the affinity of FCGR (Nagelkerke et al., 2019). The impact of the affinity alteration is the clinical response of the therapy. A recent study on rs396991 (−158F/V) *FCGR3A* showed a statistically significant rituximab efficacy in the V allele (wild-type) compared with FF genotype as a homozygote mutant. Rituximab was more effective in V allele carriers (94%) compared with those of the FF (81%) (Robledo et al., 2012a) (Table S2). Another cause that alteration of the gene expression could impact the clinical response of the drugs is IL expression. Results of the study on polymorphism on IL-1β proved that genetic variance related to increasing IL-1β levels might be unfavorable for the treatment of psoriatic with monoclonal antibody anti-TNFα r ustekinumab (Loft et al., 2018) (Table S2).

## 7 Limitation

This review is subject to have slight potential bias, including the influence of the author’s viewpoints, gaps in literature searching, and selection methods, that may lead to the omission of relevant research.

## 8 Challenges and Future

Personalized medicine practices are an endeavor that will yield effective-significant results in the treatment of SLE and other autoimmune diseases. SLE treatment would be based on the genetic profiles that are related to pathogenesis and response therapy. The difficulty that becomes a challenge in its implementation is the limited human and logistical resources, especially in the low-middle and developing countries.

## 9 Conclusion and Prospects

SLE is a disease that is closely related to genetic factors, not only the pathology but also the response of therapy. A polymorphism that occurs on the genes related to drugs pharmacokinetics or drug-target pharmacodynamics causes clinical response variation of the drugs. Has become a must-to-do personalized medicine for the patients, providing an individual therapy based on genetic profile. It becomes necessary because it gives better and more effective treatments for SLE and other autoimmune disease patients.
