# Supplementary material for: Genetic Polymorphisms and the Clinical Response to Systemic Lupus Erythematosus Treatment Towards Personalized Medicine
Source: Front Pharmacol. 2022 Mar 18;13:820927. doi: 10.3389/fphar.2022.820927 (PMC8972168; doi:10.3389/fphar.2022.820927)
Supplement: Supplementary file 1 [file DataSheet1.docx]

Supplementary Table S1. SNPs of genes related to pharmacokinetic that may alter clinical response of drugs in SLE therapy.

| **Gene** | **Gene specific** | **SNP** | **Subject** | **Disease** | **Result** | **Conclusion** | **Drugs** | **Ref** |
| --- | --- | --- | --- | --- | --- | --- | --- | --- |
| ***CYP*** | *CYP3A5* | rs776746 | 55 subject, Asian (Japan) | SLE & RA | GG VS AA (p< 0.05) d=1.4 | GG genotype (homozygote mutant) has a higher blood concentration than the wild type | Tacrolimus | ^44^ |
|  |  |  | 194 subject, Asian (Korea) | SLE | Not significant | | Hydroxychloroquine | ^45^ |
|  |  |  | 8 subject, Asian (Japan) | RA, dermatomyositis, purpura nephritis, SLE | AA no increase of tacrolimus blood concentration. AG increase occurred in 1 patien (50%). GG increase occurred in a whole subject 100%. | AA no increase of tacrolimus blood concentration. AG increase occurred in 1 patient (50%). GG increase occurred in a whole subject 100%. | Tacrolimus | ^118^ |
|  |  |  | 100 subject, Asian (China) | Nephrotic Syndrome | do not affect the clinical efficacy of TAC | | Tacrolimus | ^34^ |
|  |  |  | 220 subject, Asian ( Indian) | Lupus Nephritis | This variance synergetic influence on CYC failure (OR=8.2 p<0.001). metabolic activity was lower in TC allele as compared with TT allele. this variance has significant decrease of AUC 0-t, C_max_, and t1/2 (p<0.02) | Patients with rs776746 have synergistic influence on CYC failure. | Cyclophosphamide | ^47^ |
|  | *CYP3A4* | rs28371759 | 194 subject, Asian (Korea) | SLE | Not significant | | Hydroxychloroquine | ^45^ |
|  |  | rs55785340 | 33 subject, Caucasian (Hungary) | Autoimmune disease (7 SLE, 21,2%) | Not available | | Cyclophosphamide | ^50^ |
|  |  | rs2740574 |  |  |  |  |  |  |
| **Gene** | **Gene specific** | **SNP** | **Subject** | **Disease** | **Result** | **Conclusion** | **Drugs** | **Ref** |
| ***CYP*** | *CYP3A4* | rs4986910 | 33 subject, Caucasian (Hungary) | Autoimmune disease (7 SLE, 21,2%) | Not available | | Cyclophosphamide | ^50^ |
|  |  | rs2242480 | 100 subject, Asian (China) | Nephrotic Syndrome | Not affect the clinical efficacy of TAC | | Tacrolimus | ^34^ |
|  | *CYP2D6* | rs1135840 | 194 subject, Asian (Korea) | SLE | DHCQ:HCQ ratio highest in CC, lowest in GG (p<0.01) | Significant associated with DCHQ: HCQ ratio. Blood concentration of HCQ related to CYP2D6 polymorphism | Hydroxychloroquine | ^45^ |
|  |  | rs1065852 |  |  | DHCQ:HCQ ratio highest in GG, lowest in AA (p=0,03) |  |  |  |
|  |  | rs3892097 | 200 subject, Caucasian (UK) | discoid lupus | Disseminated disease (OR=0.21 CI095%(0.08-0.52) p<0.01) all mutant VS WT. Concomitant SLE (OR=0.06 CI95%(0.01-0.49)). Associated with lack of HCQ response | CYP genotype polymorph did not have any significant association to HCQ response. Baseline lupus severity are predictor of response to HCQ. | Hydroxychloroquine | ^119^ |
|  | *CYP2C19* | rs424485 | 71 subject, Asian (Thailand) | SLE | Wild type allele has a highest ovarian toxicity (OR=11.0 CI 95% 1.2-99.1, a OR=13.6 CI 95% 1.1-162.2) | CYP2C19*1 genotype increase risk of ovarian toxicity 5 fold. 23.75 gram or higher dose of Cyclophosphamide carries a 2 fold risk | Cyclophosphamide | ^46^ |
|  |  |  | 220 subject, Asian ( Indian) | Lupus Nephritis | WT A allele has a better response. As an independent predictor of CYC therapeutic failure (odds ratio [OR]:  2.69; p = 0.0043) | Patients with rs4244285 were at increased risk of CYC failure | Cyclophosphamide | ^47^ |
| **Gene** | **Gene specific** | **SNP** | **Subject** | **Disease** | **Result** | **Conclusion** | **Drugs** | **Ref** |
| ***CYP*** | *CYP2C8* | rs10509681 | 200 subject, Caucasian (UK) | discoid lupus | Disseminated disease (OR=0.21 CI095%(0,08-0.52) p<0.01). Concomitant SLE (OR=0.06 CI95%(0.01-0.49)). Associated with lack of HCQ response | CYP genotype polymorph did not have any significant association to HCQ response. Baseline lupus severity are predictor of response to HCQ. | Hydroxychloroquine | ^119^ |
|  | *CYP2C9* | rs1799853 | 220 subject, Asian ( Indian) | Lupus Nephritis | Not significant (p=0.58) | Not significant | Cyclophosphamide | ^47^ |
|  |  |  | 30 subject, Asian (China) | SLE | All exposure among genotype were not statistically significant (p>0.05) | | Teriflunamide and leflunamide | ^36^ |
|  |  |  | 30 subject, Asian (China) | SLE | This SNP did not fulfil the conditions; The relative standard errors (RSE%), representing the uncertainty, for fixed-effect parameters were within 57%. The uncertainties for some random-effect parameters were <47%. | | Teriflunamide | ^37^ |
|  |  | rs1057910 | 220 subject, Asian ( Indian) | Lupus Nephritis | Not significant (p=1) | Not significant | Cyclophosphamide | ^47^ |
|  |  |  | 30 subject, Asian (China) | SLE | All exposure among genotype were not statistically significant (p>0.05) | | Teriflunamide and leflunamide | ^36^ |
|  |  |  | 30 subject, Asian (China) | SLE | This SNP did not fulfil the conditions; The relative standard errors (RSE%), representing the uncertainty, for fixed-effect parameters were within 57%. The uncertainties for some random-effect parameters were <47%. | | Teriflunamide | ^37^ |
| ***CYP*** | *CYP2B6* | rs3745274 | 33 subject, Caucasian (Hungary) | Autoimmune disease (7 SLE, 21,2%) | Not significant (p=0,62) | Not significant | Cyclophosphamide | ^50^ |
|  |  | rs2279343 |  |  | Not significant (p=0,12) | Not significant |  |  |
| **Gene** | **Gene specific** | **SNP** | **Subject** | **Disease** | **Result** | **Conclusion** | **Drugs** | **Ref** |
| ***ABC* transporter** | *ABCB1* | rs1045642 | 8 subject, Asian (Japan) | RA, dermatomyositis, purpura nephritis, SLE | No increase, not detectable (blood concentration of tacrolimus lower than threshold), treatment was not achieve. | Detection and measurable genetic polymorphism is applicable for selection immunosuppression or individual dosage for autoimmune treatment. | Tacrolimus | ^118^ |
|  |  | rs2032582 |  |  |  |  |  |  |
|  |  |  | 55 subject, Asian (Japan) | SLE & RA | Not significant | Did not show any effect | Tacrolimus | ^44^ |
|  |  |  | 100 subject, Asian (China) | Nephrotic Syndrome | Increase the effectiveness. Mutant genotype VS wild type GG (p=0.042, OR=8.638, CI 95%(1.08-69.819)) | ABCB1 is probably influencing the clinical efficacy of TAC in NS patients. | Tacrolimus | ^34^ |
|  |  | rs1128503 |  |  | Increase the effectiveness. TT vs CC/CT (p=0,018, OR=12,085, CI 95% (1,535-95,148) |  |  |  |
|  | *ABCC2* | rs2273697 | 88 subject, Asian (Hong Kong) | Lupus Nephritis | AG VS GG (p=0.003). Corresponded to higher lymphocyte count and platelet level (p=0.010). AG has no association with clinical flare | rs2273697 AG genotype is associated with lower MPA exposure. | Myophenolic acid | ^35^ |
|  |  | rs3740066 |  |  | Not significant and no association (p>0.05). | |  |  |
|  |  | rs717620 |  |  |  |  |  |  |
|  |  | rs17222723 |  |  |  |  |  |  |
| **Gene** | **Gene specific** | **SNP** | **Subject** | **Disease** | **Result** | **Conclusion** | **Drugs** | **Ref** |
| ***ABC* transporter** | *ABCG2* | rs2231142 | 30 subject, Asian (China) | SLE | On teriflunamide, AA has 70.4% lower of AUC than in wild type. On leflunamide AA has 30% lower AUC than in wild type. But not significant | *ABCG2* rs2231137 was found significance affect teriflunamide pharmacokinetic suggesting may be a influencing factor | Teriflunamide and leflunamide | ^36^ |
|  |  |  | 30 subject, Asian (China) | SLE | GA (51% higher absorption rate, 37% higher clearance). AA (97% higher absorption rate, 111% higher clearance) than wild type GG. | *ABCG2* rs2231142 identified to significant affect pharmacokinetic characteristic of teriflunamide (absorption and clearance). | Teriflunamide | ^37^ |
|  |  | rs2231137 | 30 subject, Asian (China) | SLE | All exposure among genotype were not statistically significant (p>0.05) | | Teriflunamide and leflunamide | ^36^ |
|  |  |  | 30 subject, Asian (China) | SLE | This SNP did not fulfil the conditions; The relative standard errors (RSE%), representing the uncertainty, for fixed-effect parameters were within 57%. The uncertainties for some random-effect parameters were <47%. | | Teriflunamide | ^37^ |
| ***GST*** | *GSTP1* | rs1695 | 220 subject, Asian ( Indian) | Lupus Nephritis | This variance synergetic influence on CYC failure (OR=8.2 p<0.001). AA genotype has a better response (OR: 2.8 CI%(1.15-16.9) p=0.03 | rs1695 have synergistic influence on CYC failure. | Cyclophosphamide | ^47^ |
|  |  |  | 33 subject, Caucasian (Hungary) | Autoimmune disease (7 SLE, 21,2%) | Wild type = 21.42%. Heterozygote=61.5%, homozygote=50%. to disease remission (response) p=0.03 | Decrease activity of *GSTP1* can be background of the more effective disease treatment | Cyclophosphamide | ^50^ |
| **Gene** | **Gene specific** | **SNP** | **Subject** | **Disease** | **Result** | **Conclusion** | **Drugs** | **Ref** |
| ***GST*** |  |  | 95 subject, Asian (Myanmar) | Lupus Nephritis | No significance differences in myelotoxicity (p=1), leukopenia (p=0.61), thrombocytopenia (p=1). No significances in remission of the disease in 3 months (p=0.62) and in 6 months (p=0.589) | No significances differences between each of genotype group with regard in remission or myelotoxicity. | Cyclophosphamide | ^120^ |
|  | *GSTT1* | rs17856199 | 220 subject, Asian ( Indian) | Lupus Nephritis | There were no signiﬁcant differences in the pharmacokinetic parameters of CYC with the studied variants | | Cyclophosphamide | ^47^ |
|  |  |  | 33 subject, Caucasian (Hungary) | Autoimmune disease (7 SLE, 21,2%) | Not significant (p=0.41) | Not significant | Cyclophosphamide | ^50^ |
|  | *GSTM1* | rs366631 | 220 subject, Asian ( Indian) | Lupus Nephritis | There were no signiﬁcant differences in the pharmacokinetic parameters of CYC with the studied variants | | Cyclophosphamide | ^47^ |
|  |  |  | 33 subject, Caucasian (Hungary) | Autoimmune disease (7 SLE, 21,2%) | Not significant (p=0.88) | Not significant | Cyclophosphamide | ^47^ |
|  | *GSTA1* | rs3957356 | 94 subject, Asian ( Egypt) | Lupus Nephritis | TT vs CT/CC associated to CYC resistance and partial response (p=0.033). None of TT genotype has complete remission (p=0.047). CYC related side effect (CC vs TT, p=0.03. CT vs TT, p=0.0199). | Lupus Nephritis with *GSTA1* TT genotype have a highest risk of CYC unresponsiveness and toxicity | Cyclophosphamide | ^51^ |
| ***OATP*** | *OATPs* | rs7311358 | 88 subject, Asian (Hongkong) | Lupus Nephritis | Not significant and no association (p>0.05). | Not significant | Myophenolic acid | ^35^ |
|  |  | rs4149117 |  |  |  |  |  |  |
| **Gene** | **Gene specific** | **SNP** | **Subject** | **Disease** | **Result** | **Conclusion** | **Drugs** | **Ref** |
| ***UGT*** | *UGT* | rs17863762 | 88 subject, Asian (Hong Kong) | Lupus Nephritis | Had the same genotype, no variance | | Myophenolic acid | ^35^ |
|  |  | rs6714486 |  |  |  |  |  |  |
|  |  | rs17868320 |  |  |  |  |  |  |
|  |  | rs72551330 |  |  |  |  |  |  |
| ***TPMT*** | *TPMT* | rs1142345 | 250 subject, Asian (Bangladesh) | SLE | Mutant vs wildtype genotype: OR=17.6 CI95% (5.8-53.6) p= 0.0001 severe leukopenia (grade III/IV). OR= 13.4 (4.6-39.2) severe thrombocytopenia (grade III/IV) p=0.0001. | SLE with rs1142345 polymorphism (heterozygote) at a risk of azathioprine induced myelosuppression | Azathioprine | ^65^ |
|  |  | rs1800460 |  |  | No patients carried a homozygous mutation or the *TPMT*3B* or *TPMT*2* polymorphisms. |  |  |  |
|  |  | rs1800462 |  |  |  |  |  |  |
| ***NAT*** | *NAT1* | rs15561 | 30 subject, Asian (China) | SLE | Other SNP did not fulfil the conditions; The relative standard errors (RSE%), representing the uncertainty, for fixed-effect parameters were within 57%. The uncertainties for some random-effect parameters were <47%. | Not available | Teriflunamide | ^37^ |
|  |  | rs1057126 |  |  |  |  |  |  |
|  |  | rs4986980 |  |  |  |  |  |  |
|  |  | rs4986989 |  |  |  |  |  |  |
|  |  | rs4987076 |  |  |  |  |  |  |
|  |  | rs4986782 |  |  |  |  |  |  |
|  |  | rs56379106 |  |  |  |  |  |  |
| ***NQO*** | *NQO1* | rs689459 | 114 subject, negroid (Africa) and Caucasian (Europe) | Inflammatory and autoimmune disease | eQTL at a FDR<0.1 play an important roles in GC related biological processes. | identified interactions that influence the expression of genes known to play central roles in GC-related pathways | Glucocorticoid | ^67^ |

Supplementary Table S2. SNPs of genes related to pharmacodynamic mechanism that may alter clinical response of drugs in SLE therapy.

| **Gene** | **Gene** | **SNP** | **Subject** | **Disease** | **Result** | **Conclusion** | **Drugs** | **Ref** |
| --- | --- | --- | --- | --- | --- | --- | --- | --- |
| ***FCGR*** | *FCGR3A* | rs10127939 | 159 subject, Caucasian (USA) | Waldenstrom Macroglobulinemia | P=0.03, 44.4% CR on LH or LR alleles | Associate with improvement in clinical response and longer PFS | Rituximab | ^72^ |
|  |  | rs396991 |  |  | p=0.008, 77.8% CR on at least one V alleles |  |  |  |
|  |  |  | 70 subject, Caucasian (Barcelona, Spain) | Psoriasis | FCGR high affinity (VV158, VF158) had a lower mean intermediate BSA than did patients presenting only low affinity alleles (p=0,03 and p =0,02). associated to greater reduction of BSA. | FCGR3A has implication to outcome of anti TNF-α in psoriasis treatment | Anti TNF- α | ^71^ |
|  |  |  | 132 subject, Caucasian (Spain) | Systemic autoimmune disease (83 SLE, 57,6%) | V allele = 38% responder, 16% non-responder (p=0.01, OR=3.24, CI. 95% 1.17-11.1). V allele has a better response (V allele carrier: homozygote = 94%:81%). P=0.02, OR=3.96, CI 95% (1.1-17.68) | rs396991 play a role in clinical response of rituximab in autoimmune disease treatment | Rituximab | ^121^ |
|  |  |  | 214 subject, Caucasian (Sweden) | RA | V allele related to LON 4 fold (p=0.017), 158V/V associated with longer flare free survival (p=0.031) and LON (p=0.023) | *FCGR3A* V allele could be an indicator a biological therapeutic activity through LON incidence and longer flare free survival. | Rituximab | ^69^ |
|  |  |  | 16 articles | SLE | VV/FF RR= 1.13 CI95% (0.91-1.39) p=0.25. FV/VV RR=1,04 CI95% (0.87-1.24) p=0.687. FV/FF RR=1.17 (0.99-1.37) p=0.56 | A number of potential predictor should be validated as being specific for response to RTX therapy | Rituximab | ^122^ |
| **Gene** | **Gene** | **SNP** | **Subject** | **Disease** | **Result** | **Conclusion** | **Drugs** | **Ref** |
| ***FCGR*** | *FCGR2A* | rs201218628 | 159 subject, Caucasian (USA) | Waldenstrom Macroglobulinemia | Not associated | | Rituximab | ^72^ |
|  |  | rs1801274 |  |  |  |  |  |  |
|  |  |  | 214 subject, Caucasian (Sweden) | RA | Not associated | | Rituximab | ^69^ |
|  |  |  | 70 subject, Caucasian (Barcelona, Spain) | Psoriasis | FCGR high affinity (HH131, HR131 had a lower mean intermediate BSA than did patients presenting only low affinity alleles (p=0.03 and p =0.02). associated to greater reduction of BSA. | FCGR2A has implication to outcome of anti TNF-α in psoriasis treatment | Anti TNF-α | ^71^ |
|  | *FCGR2B* | rs1050501 | 159 subject, Caucasian (USA) | Waldenstrom Macroglobulinemia | Not associated | | Rituximab | ^72^ |
|  |  |  | 214 subject, Caucasian (Sweden) | RA | Not associated | | Rituximab | ^69^ |
| ***BAFF*** | BAFF promoter | rs9514828 | 214 subject, Caucasian (Sweden) | RA | CT genotype has tendency longer free survival (T/T allotype) (p=0.0096) | The role of BAFF promoter polymorphism in LON is unclear. | Rituximab | 9 |
| ***Interleukin*** | *IL1B* | rs4848306 | 738 subject, Caucasian (Denmark) | Crohn’s Disease, Ulcerative Colitis | Response (GA or AA) 1.85 (1.05–3.27) | the cytokines IL-1b, may be potential targets for treating patients with IBD who do not respond to anti-TNF therapy | Anti TNF-α | ^77^ |
| **Gene** | **Gene** | **SNP** | **Subject** | **Disease** | **Result** | **Conclusion** | **Drugs** | **Ref** |
| ***Interleukin*** |  | rs1143623 | 478 subject, Caucasian (Denmark) | Psoriatic | Associated to clinical response of anti TNF-α and ustekinumab (q<0.2) | Genetic variance related to increase IL-1beta levels may be unfavourable when treat psoriatic with anti TNF-α or ustekinumab | Anti TNF-α | ^78^ |
|  |  | rs143627) |  |  |  |  |  |  |
|  | *IL1RN* | rs4251961 | 738 subject, Caucasian (Denmark) | Crohn’s Disease, Ulcerative Colitis | Nonresponse (TC or CC) 0.42 (0.18–0.98) | the cytokines, may be potential predictor for clinical response of anti TNF-alpha | Anti TNF-α | ^77^ |
|  | *IL6* | rs10499563 |  |  | Response (TC or CC) 2.26 (1.18–4.32) |  |  |  |
|  |  | rs10225286 | 114 subject, negroid (Africa) and Caucasian (Europe) | Inflammatory and autoimmune disease | Significant association with secretion of IL6 and affect the GC response (0=1.9x10-4) | genetic variance influence the expression of genes known to play central roles in GC-related pathways and that influence the secretion of IL6. | Glucocorticoid | ^67^ |
|  |  | rs1800795 | 144 subject, Caucasian ( Spain) | SLE, SS, IM, ANC vasculitis, Sjorgen ‘s syndrome, RA, autoimmune haemolytic anaemia. (83 SLE, 57,6%) | Cc genotype (non-responder =23.5%; responder =7.1%). P=0.049, OR =4.03, CI 95% (0.78-16.97). Effectiveness of RTX at a glance CC (69.2%) GC (91.9%) GG (88.4%). In SLE, CC genotype (non-responder = 27.3%, responder=6.9%). P=0.066, OR=5.1. CI 95% (0.65-31.73). Effectiveness of RTX in SLE CC (62.5%) GC (88.9%) GG (90%). | rs1800795 play a role in rituximab clinical response | Rituximab | ^121^ |
| **Gene** | **Gene** | **SNP** | **Subject** | **Disease** | **Result** | **Conclusion** | **Drugs** | **Ref** |
| ***Interleukin*** | *IL6* | rs1800795 | 16 articles | SLE | GG/GC (90%: 88.9%) RR=1.01 CI 95% (0.87-1.18) p=0.87. GG/CC (90%:62.5%) RR=1.26 CI95% (0.77-2.03) p=0.34. GC/CC (88.9%:62.5%) RR=1.42 CI95% (0.82-2.46) p=0.4 | A number of potential predictor should be validated as being specific for response to RTX therapy | Rituximab | ^122^ |
|  | *IL17A* | rs2275913 | 738 subject, Caucasian (Denmark) | Crohn’s Disease, Ulcerative Colitis | Nonresponse (GA or AA) 0.42 (0.18–1.00) | the cytokines, may be potential predictor for clinical response of anti TNF-α | Anti TNF-α | ^77^ |
|  | *IL2-IL21* | rs6822844 | 84 subject and 60 subject, Caucasian (Spain) | SLE and other systemic autoimmune disease (84 SLE, 58%) | GG in SLE (responder = 83.56%, non-responder = 45.45%). P=0.01 OR=6.1 CI 95% (1.28-29.06). At general pooled analysis p=0.024 OR= 3.53 CI95%(1.06-11.64). | rs6822844 play a role in response to rituximab for treating SLE | Rituximab | ^79^ |
|  |  |  | 16 articles | SLE | GG/GT RR=1.39 (0.99-1.94) p= 0.055, OR=6.43 (1.42-21.07) p=0.016 | Prognostic factor cannot be used to make any specific recommendation for clinical practice. A number of potential predictor should be validated as being specific for response to RTX therapy | Rituximab | ^122^ |
|  | *IL10* | rs1800896 | 386 subject, Caucasian (Spain) | SLE | Genotype with low affinity (AA/AG) has significant effect (p=0.005) | *IL10* polymorphism influence the antimalarial regulation (being down regulated) | Antimalarial | ^80^ |
| **Gene** | **Gene** | **SNP** | **Subject** | **Disease** | **Result** | **Conclusion** | **Drugs** | **Ref** |
| *IFNγ* | *IFNγ* | rs2430561 | 738 subject, Caucasian (Denmark) | Crohn’s Disease, Ulcerative Colitis | Response (TA or AA) 1.66 (1.05–2.62) | the cytokines, may be potential predictor for clinical response of anti TNF- α | Anti TNF-α | ^77^ |
|  |  | rs2430561 | 140 subject, Asian (Iran) | Immune thrombocytopenia | No statistically significant association between platelet count and genotype. P=0.74 before treatment, p=0.9 after treatment | Does not seem to have any role in ITP pathogenesis and treatment response | Prednisone (iv) | ^123^ |
| *TLR* | *TLR2* | rs4696480 | 738 subject, Caucasian (Denmark) | Crohn’s Disease, Ulcerative Colitis | Nonresponse (TT) 0.47 (0.23–0.95) | polymorphisms in genes involved in activating NFkB through the Toll-like receptor (TLR) pathways, are important predictors for the response to anti-TNF therapy | Anti TNF-α | ^77^ |
|  |  | rs11938228 |  |  | Nonresponse (CA or AA) 0.63 (0.41–0.98) |  |  |  |
|  |  |  | 478 subject, Caucasian (Denmark) | Psoriatic | Associated to clinical response of anti TNF-α (q<0.2) | were associated with response to anti TNF- α treatment | Anti TNF-α | ^78^ |
|  |  | rs1816702 | 738 subject, Caucasian (Denmark) | Crohn’s Disease, Ulcerative Colitis | Response (CT or TT) 2.02 (1.04–3.95) | polymorphisms in genes involved in activating NFkβ through the TLR pathways, are important predictors for the response to anti-TNF therapy | Anti TNF-α | ^77^ |
|  |  | rs3804099 |  |  | Response (TC or CC) 1.80 (1.15–2.81) |  |  |  |
| **Gene** | **Gene** | **SNP** | **Subject** | **Disease** | **Result** | **Conclusion** | **Drugs** | **Ref** |
| *TLR* |  | rs4676480) | 478 subject, Caucasian (Denmark) | Psoriatic | Associated to clinical response of anti TNF-α (q<0.2) | Were associated with response to anti TNF-α treatment | Anti TNF-α | ^78^ |
|  | *TLR4* | rs5030728 | 738 subject, Caucasian (Denmark) | Crohn’s Disease, Ulcerative Colitis | Response (GA or AA) 1.45 (1.06–2.00) | polymorphisms in genes involved in activating NFkB through the Toll-like receptor (TLR) pathways, are important predictors for the response to anti-TNF therapy | Anti TNF-α | ^77^ |
|  |  | rs1554973 |  |  | Nonresponse ( TC or CC) 0.72 (0.52–0.99) |  |  |  |
|  | *TLR5* | rs5744174 | 478 subject, Caucasian (Denmark) | Psoriatic | Associated to clinical response of ustekinumab (q<0.2) | were associated with response to ustekinumab | Anti TNF-α | ^78^ |
|  | *TLR9* | rs187084 | 738 subject, Caucasian (Denmark) | Crohn’s Disease, Ulcerative Colitis | Response ( TC) 1.99 (1.04–3.82) | polymorphisms in genes involved in activating NFkB through the Toll-like receptor (TLR) pathways, are important predictors for the response to anti-TNF therapy | Anti TNF-α | ^77^ |
|  |  | rs352139 |  |  | Nonresponse (AA) 0.48 (0.24–0.96) |  |  |  |
|  |  |  | 478 subject, Caucasian (Denmark) | Psoriatic | Associated to clinical response of anti TNF-α (q<0.2) | were associated with response to anti TNF-α treatment | Anti TNF-α | ^78^ |
| *LY* | *LY96* | rs11465996 | 738 subject, Caucasian (Denmark) | Crohn’s Disease, Ulcerative Colitis | Response (CG or GG) 1.48 (1.00–2.19) | polymorphisms in genes involved in activating NFkB, are important predictors for the response to anti-TNF therapy | Anti TNF-α | ^77^ |
| *CD* | *CD14* | rs2569190 |  |  | Nonresponse (GA or AA) 0.54 (0.30–0.98) |  |  |  |
| *MAPK3* | *MAP3K14* | rs7222094 |  |  | Response ( TC) 1.92 (1.00–3.68) |  |  |  |
| *TNF* | *TNFA* | rs361525 |  |  | Nonresponse (GA) 0.43 (0.19–0.97) |  |  |  |
| **Gene** | **Gene** | **SNP** | **Subject** | **Disease** | **Result** | **Conclusion** | **Drugs** | **Ref** |
| *TNF* |  | rs361525 | 112 subject, Caucasian | psoriatic arthritis | No significance | | Anti TNF-α | ^124^ |
|  |  | rs1800629 |  |  |  |  |  |  |
|  |  |  | 386 subject, Caucasian (Spain) | SLE | Genotype with high affinity (AA/AG) has significant effect (p=0.001) | TNF-α promoter and IL10 polymorphism influence the anti-malarial being down regulated | Antimalarial | ^80^ |
|  |  | rs80267959 | 112 subject, Caucasian | psoriatic arthritis | Rs80267959 With susceptibility disease (p=0.0136), with response (p=0.021) | rs80267959 shows association with response to etanercept | Anti TNF-α | ^124^ |
|  | *TNFAIP3* | rs6927172 | 738 subject, Caucasian (Denmark) | Crohn’s Disease, Ulcerative Colitis | Nonresponse (CG or GG) 0.62 (0.42–0.92) | genes regulating NFkB signalling important were predictors for the response to anti-TNF therapy | Anti TNF-α | ^77^ |
|  |  | rs6870205 | 114 subject, negroid (Africa) and Caucasian (Europe) | Inflammatory and autoimmune disease | was significantly associated with patient response (p=2.5x10-3) | identified interactions that influence the expression of genes known to play central roles in GC-related pathways and that influence the secretion of IL6. | Glucocorticoid | ^67^ |
|  |  | rs2230926 | 199 subject, Caucasian (USA) | Psoriasis | G allele similarly associated like rs610604 | Significant correlation between polymorphism against TNF-α blocker on clinical response in psoriasis patients. | Anti TNF-α | ^125^ |
|  |  | rs610604 |  |  | G allele has significant association with response of TNF-α blocker (OR=1.5, p=0.05) etanercept (OR=1.64 p=0.016). |  |  |  |
| *TNFRs* | *TNFRSF1A* | rs4149570 | 738 subject, Caucasian (Denmark) | Crohn’s Disease, Ulcerative Colitis | Response (TT) 2.07 (1.03–4.15) | genes regulating TNF-α signalling important were predictors for the response to anti-TNF therapy | Anti TNF-α | ^77^ |
| **Gene** | **Gene** | **SNP** | **Subject** | **Disease** | **Result** | **Conclusion** | **Drugs** | **Ref** |
| *TNFRs* | *C1Orf106* | rs4915463 | 114 subject, negroid (Africa) and Caucasian (Europe) | Inflammatory and autoimmune disease | Local effect on GC significant, log fold change (FDR>0.67, p=8.4x10-11). | identified interactions that influence the expression of genes known to play central roles in GC-related pathways | Glucocorticoid | ^67^ |
|  | *C9Orf5* | rs10816772 |  |  | Validation of regulatory polymorphism with treatment-specific effect (p=0,041), affect up-regulated genes (p=6.8x10-3) |  |  |  |
| *Tirap* | *TIRAP* | rs8177374 | 478 subject, Caucasian (Denmark) | Psoriatic | Associated to clinical response of ustekinumab (q<0.2) | were associated with response to ustekinumab | Anti TNF Alfa | ^78^ |
| *AIRE* | *AIRE* | rs762421 | 114 subject, negroid (Africa) and Caucasian (Europe) | Inflammatory and autoimmune disease | Was associated with risk of Crohn’s disease in GC treatment | identified interactions that influence the expression of genes known to play central roles in GC-related pathways | Glucocorticoid | ^67^ |
| *GR* | *GR* | rs4912905 | 220 subject, Asian (China) | SLE | Dominant genotype OR=0.41 CI95% (0.23-0.72) p=0.02. aOR=0.419 CI95% (0.23-0.75) p=0.04. | *GR* gene polymorphism plays a role in GC response for SLE | Glucocorticoid | ^15^ |
|  |  | rs17100234 |  |  | Dominant genotype OR= 0.52 CI95% (0.282-0.96) p=0.038. aOR= 0.52 CI95% (0.779-0.97) p=0.04 |  |  |  |
|  |  | rs7701443 |  |  | Recessive genotype OR= 2.736 CI95% (1.183-6.331) p=0.019. aOR=2.639 CI95% (1.16-6.23) p=0.027 |  |  |  |
| **Gene** | **Gene** | **SNP** | **Subject** | **Disease** | **Result** | **Conclusion** | **Drugs** | **Ref** |
| *HSP* | *HSP90B1* | rs12426382 | 291 subject, Asian (China) | SLE | GC efficacy associated to dominant genotype (P=0.006, OR=0.514 CI95% (0.321-0.824) aP=0.007 aOR=0.513 CI95% (0.317-0.831) afrer BH correction = no association. CC associated with efficacy GC, p=0.048 (after BH). OR=0.436 CI95% (0.208-0.916) p=0.025 | SNP in *HSP90B1* gene might be associated with efficacy GC, but not related to HRQOL | Glucocorticoid | ^93^ |
|  |  | rs3794241 |  |  | polymorphisms were associated with the improvement of HRQoL among SLE patients (P < 0.05). But no association existed after the correction of BH method (P > 0.05) |  |  |  |
|  |  | rs1165681 |  |  |  |  |  |  |
|  |  | rs2722188 |  |  |  |  |  |  |
|  |  | rs3794240 |  |  |  |  |  |  |
|  |  | rs10861147 |  |  |  |  |  |  |
| *TRAP* | *TRAP1* | rs8055172 | 958 subject, Asian (China) | SLE | Associated to susceptibility SLE (p=3.54x10-7) in dominant model | TRAP1 may be associated with efficacy of GC | Glucocorticoid | ^96^ |
|  |  | rs2072379 |  |  | related to oral ulcers (p = 0.021) |  |  |  |
|  |  | rs8055172 |  |  | TT genotype associated to susceptibility of SLE (p = 4.26×10–4) |  |  |  |
|  |  | rs1639150 |  |  | In dominant model may be associated with fever of SLE patients (p = 0.028) |  |  |  |
|  |  | rs2072379 |  |  | TC genotype associated to susceptibility of SLE (p = 6.93×10–9) |  |  |  |
|  |  | rs12597773 |  |  | Related to haematological system (p = 0.035) |  |  |  |
|  |  | rs6500552 |  |  | Associated to efficacy of GC (p=0,004) in dominant model |  |  |  |
| **Gene** | **Gene** | **SNP** | **Subject** | **Disease** | **Result** | **Conclusion** | **Drugs** | **Ref** |
| TRAP | TRAP1 | rs3794701 |  |  | was associated with the improvement in role-emotional (RE) of SLE patients in dominant model (p = 0.029) | TRAP1 may be associated with efficacy of GC | Glucocorticoid | ^96^ |
|  |  | rs3751842 |  |  | In dominant model may be associated with fever of SLE patients (p = 0.035) |  |  |  |
